# BACE1 Inhibition Protects Against Type 2 Diabetes Mellitus by Restoring Insulin Receptor in Mice

**DOI:** 10.3390/ijms26115100

**Published:** 2025-05-26

**Authors:** Tingting Lin, Ting Liang, Yong Shen, Feng Gao

**Affiliations:** 1 Neurodegenerative Disorder Research Center, Division of Life Sciences and Medicine, University of Science and Technology of China, Hefei 230026, China; ltt5@mail.ustc.edu.cn (T.L.); tingliang@mail.ustc.eu.cn (T.L.); 2 Anhui Province Key Laboratory of Biomedical Aging Research, University of Science and Technology of China, Hefei 230001, China

**Keywords:** β-secretase 1 (BACE1), insulin receptor (InsR), insulin resistance, type 2 diabetes mellitus (T2DM), cognitive impairment

## Abstract

β-secretase 1 (BACE1), known for its role in amyloid-β production associated with Alzheimer’s disease (AD), has also been suggested to be elevated in patients with Type 2 diabetes mellitus (T2DM). Notably, BACE1 could cleave the insulin receptor (InsR), leading to reduced InsR levels, which may impair insulin signaling and contribute to insulin resistance. Presently, we observed decreased InsR levels and impaired glucose disposal in the livers of mice with systemic overexpression of BACE1 (HUBC mice). This suggests that elevated BACE1 could contribute to insulin resistance by shedding membrane InsR. Additionally, mice fed a high-fat diet (HFD), a well-established model of T2DM, displayed increased BACE1 levels and decreased InsR. To further investigate whether inhibiting BACE1 could enhance insulin sensitivity and alleviate symptoms of diabetes, we treated HFD mice with the BACE1 inhibitor Elenbecestat. Remarkably, the administration of Elenbecestat restored InsR levels and improved their downstream signaling pathways, leading to increased insulin sensitivity and enhanced glucose tolerance. In summary, our findings suggest that inhibiting BACE1 can restore InsR expression and improve insulin-signaling sensitivity, ultimately resulting in enhanced diabetic phenotypes.

## 1. Introduction

Diabetes mellitus affects approximately 500 million individuals globally [1], and type 2 diabetes mellitus (T2DM) accounts for over 90% of all cases [2]. A key pathological feature of T2DM is insulin-signaling dysfunction, which is relevant to the level of insulin receptor (InsR) and its downstream signaling [3,4]. InsR is a type 2 receptor tyrosine kinase that could activate the downstream IRS-PI3K-AKT pathway, playing essential roles in regulates glucose metabolism by phosphorylating various downstream proteins, which is closely associated with insulin resistance and diabetes mellitus [5,6,7,8,9,10]. Previous studies indicate a decreased InsR level in islets and acinar tissues of T2DM patients and diabetic animal models [4,11,12]. Meanwhile, InsR knockout mice exhibit characteristics of T2DM, exhibiting impaired insulin signaling, which can be significantly ameliorated by restoring the InsR level in the liver [13,14]. As such, abnormalities in InsR or insulin pathway may contribute to T2DM pathologies.

β-secretase 1 (BACE1) has been shown to contribute to Alzheimer’s disease (AD) through its cleavage on amyloid precursor protein (APP) [15]. Interestingly, similar features, like insulin resistance in the brain and peripheral tissues, were found both in AD and T2DM [16]. It is noticed that the β subunit of InsR (InsRβ) is another substrate of BACE1 [17]. The BACE1 mediated cleavage of InsRβ releases InsR from the cell membrane, which would interfere with the insulin-signaling pathway, and a clinical cohort study shows elevated plasma BACE1 level and enzyme activity of individuals with T2DM [18]. Moreover, BACE1 inhibition has been found to increase glucose uptake in muscle tissue [16,19,20]. Consistently, under the high-fat diet (HFD) which causes obesity and diabetes, BACE1 knockout could improve glucose metabolism of mice and protect mice from obesity [21]. These studies suggest that the elevated BACE1 may interfere with insulin signaling by its cleavage on InsRβ, which may contribute to insulin-signaling dysfunction in T2DM.

To investigate this hypothesis, we confirmed the increased BACE1 level in wild-type (WT) with HFD treatment (WT-HFD), which serve as a relevant animal model for T2DM. The BACE1-overexpressing mice displayed decreased InsR levels, increased adiposity, impaired glucose disposal and heightened susceptibility to obesity compared to WT mice, under both normal chow diet (NCD) and HFD. Moreover, a treatment with the BACE1 inhibitor Elenbecestat effectively restored InsR level and its downstream signaling pathway, enhanced insulin sensitivity and glucose tolerance, ultimately alleviated the diabetic pathologies and improved cognition ability in WT-HFD mice. These findings highlight the role of BACE1-mediated InsR cleavage in T2DM and suggest that targeting BACE1 may be a promising therapeutic strategy for T2DM.

## 2. Results

### 2.1. Elevated BACE1 Protein Levels and Enzyme Activity in Liver and Brain Form WT-HFD Mice

**Figure 1:**
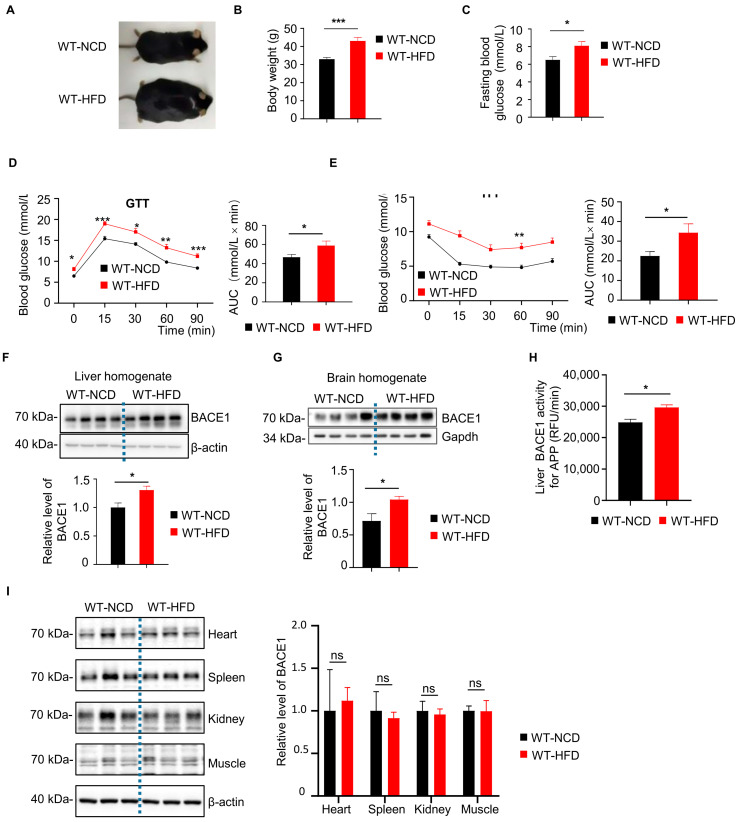
Increased β-secretase 1 (BACE1) levels and enzymatic activity in the liver and brain from wild type mice with high fat diet (WT-HFD mice) or normal chow diet (WT-NCD). (**A**) Images of WT-NCD and WT-HFD mice. (**B**) Body weight of WT-NCD and WT-HFD mice after 6 months of feeding (n = 16, 16). (**C**) Fasting blood glucose of WT-NCD and WT-HFD mice after 6 months of feeding (n = 24, 24). (**D**) Glucose tolerance tests (GTT) after 6 months of feeding and total glucose excursions during GTTs (n = 24, 24). (**E**) Insulin tolerance tests (ITT) after 6 months of feeding and total glucose excursions during ITTs (n = 24, 24). (**F**) Western blot images showing BACE1 levels in the liver from WT-NCD and WT-HFD mice; the relative level of BACE1 was normalized with β-actin (n = 8, 8). (**G**) Western blot images showing BACE1 levels in brain from WT-NCD and WT-HFD mice; the relative level of BACE1 was normalized with β-actin (n = 8, 8). (**H**) BACE1 enzymatic activity for the amyloid precursor protein (APP) substrate in the liver from WT-NCD and WT-HFD mice (n = 7, 8). (**I**) Representative western blot images of BACE1 in different tissue from WT-NCD and WT-HFD mice; the relative level of BACE1 was normalized with β-actin (n = 6, 6, 6). Data are represented as mean ± SEM. (**B**) Mann–Whitney test. (**C**), (**D**, right), (**E**, right), (**F**–**I**) Unpaired *t* test with Welch’s correction. (**D**, left) and (**E**, left) Mixed-effects analysis followed by Sidak’s multiple comparisons test. * *p* ˂ 0.05, ** *p* ˂ 0.01, *** *p* ˂ 0.001, ns means no significant differences.

We choose HFD mice as a type 2 diabetes mice model and examine the diabetic pathologies in these mice. After 6 months of HFD feeding, both body weight and fasting blood glucose level of WT-HFD mice were significantly higher than the WT-NCD group (Figure 1A–C). Also, the glucose metabolism and insulin sensitivity of mice were assessed by glucose tolerance test (GTT) and insulin tolerance test (ITT), respectively. The blood glucose levels at 0, 15, 30, 60, 90 min and the area under the curve (AUC) in GTT and ITT were higher in the WT-HFD mice than those in the WT-NCD group (Figure 1D,E), indicating the insulin resistance and impaired glucose intolerance. Interestingly, we observed that the BACE1 level was significantly increased in the liver and brain of the WT-HFD group, as compared to that in the WT-NCD group (Figure 1F,G). Meanwhile, the BACE1 cleavage activity on APP in the liver of HFD mice was also significantly increased by approximately 20% (Figure 1H). However, we did not observe any increases in BACE1 levels in the heart, spleen, kidney or muscle tissues from WT-HFD mice (Figure 1I).

### 2.2. BACE1 Overexpression Caused Reduced Liver InsR Level and Diabetic Phenotypes in HUBC Mice

**Figure 2:**
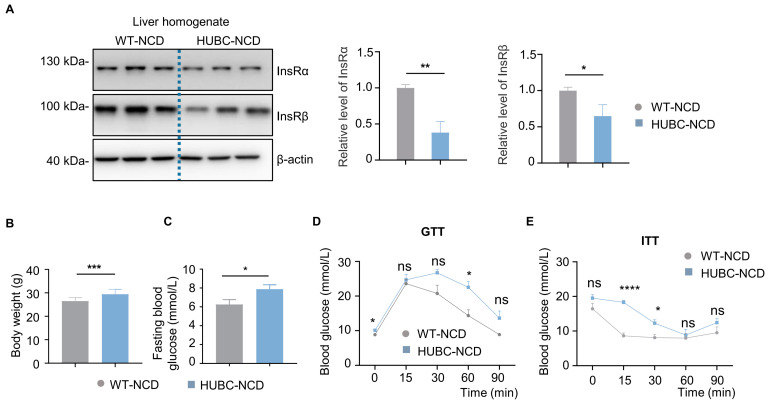
Reduced liver insulin receptor (InsR) levels and diabetic phenotypes in universal BACE1 overexpression mice with normal chow diet (HUBC-NCD). (**A**) Western blot images showing Insulin receptor subunit α (InsRα) and Insulin receptor subunit β (InsRβ) levels in liver from HUBC-NCD and littermate WT-NCD mice; the relative level of InsRβ was normalized with β-actin (n = 7, 6). (**B**) Body weight of HUBC and littermate WT mice in 6 months (n = 10, 12). (**C**) Fasting blood glucose of HUBC-NCD and littermate WT-NCD mice in 6 months (n = 10, 12). (**D**) GTTs of HUBC-NCD and littermate WT-NCD mice in 6 months (n = 10, 8). (**E**) ITTs of HUBC-NCD and littermate WT-NCD mice in 6 months (n = 10, 8). Data are represented as mean ± SEM. (**A**–**C**) Unpaired *t* test with Welch’s. (**D**,**E**) Mixed-effects analysis followed by Sidak’s multiple comparisons test. * *p* ˂ 0.05, ** *p* ˂ 0.01, *** *p* ˂ 0.001, **** *p* ˂ 0.0001. ns means no significant differences.

To further investigate the effects of the elevated BACE1, we constructed a universal BACE1 overexpression mice model (HUBC mice). Insulin signaling plays an important role in the development of type 2 diabetes, and the insulin receptor has been indicated as a substrate of BACE1 [5,17]. In HUBC mice, we observed a significant decrease in InsRβ and InsRα levels in the liver comparing to WT controls (Figure 2A). Additionally, the body weight and fasting blood glucose level of HUBC mice were significantly increased by over 18% compared to WT mice (Figure 2B,C). Meanwhile, HUBC mice showed higher glucose levels at 60 min in DTT and 15, 30 min ITT after glucose or insulin treatment, respectively, when compared to WT mice (Figure 2D,E).

**Figure 3:**
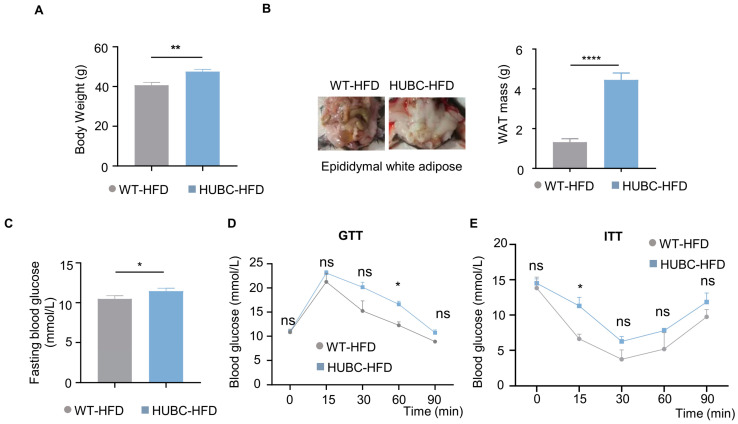
Elevated BACE1 deteriorated glucose tolerance and insulin sensitivity in HFD treatment. (**A**) Body weight of HUBC-HFD and littermate WT-HFD mice (n = 6, 6). (**B**) Represented images and mass of epididymal white adipose tissue (WAT) of HUBC-HFD and littermate WT-HFD mice (n = 6, 6). (**C**) Fasting blood glucose of HUBC-HFD and littermate WT-HFD mice (n = 6, 6). (**D**) GTTs of HUBC-HFD and littermate WT-HFD mice (n = 5, 5). (**E**) ITTs of HUBC-HFD and littermate WT-HFD mice (n = 5, 6). Data are represented as mean ± SEM. (**A**–**C**) Unpaired *t* test with Welch’s. (**D**,**E**) Mixed-effects analysis followed by Sidak’s multiple comparisons test. * *p* ˂ 0.05, ** *p* ˂ 0.01, **** *p* ˂ 0.0001, ns means no significant differences.

Under HFD treatment, HUBC mice showed a 15% increase in body weight (Figure 3A), and about a two-fold elevation of epididymal white adipose mass (WAT) compared to WT-HFD mice (Figure 3B). Meanwhile, an increased intendancy of fasting blood glucose level was also observed in HUBC-HFD mice (Figure 3C). HUBC-HFD mice showed higher blood glucose concentration at 60 min in GTT and 15 min in ITT (Figure 3D,E). Taken together, these data indicate that BACE1 overexpression can cause liver InsR reduction and diabetic phenotypes.

### 2.3. Elenbecestat Treatment Improved Glucose Tolerance and Insulin Sensitivity in HFD Mice

**Figure 4:**
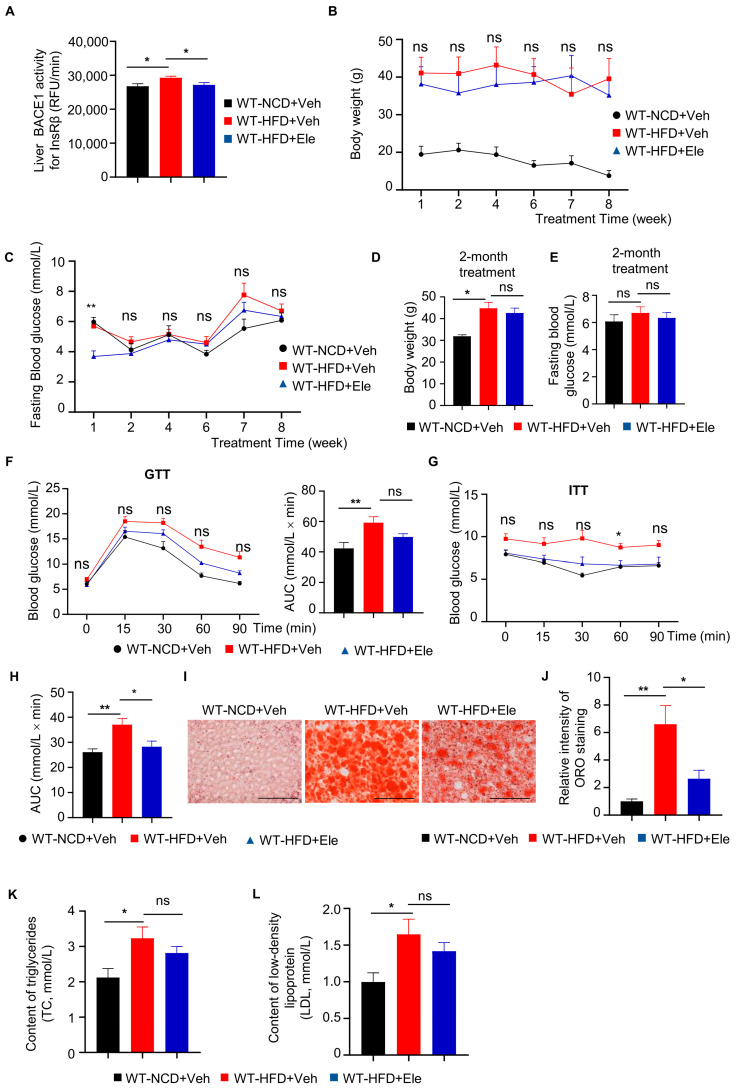
Elenbecestat treatment improved diabetic phenotypes in HFD mice. (**A**) BACE1 enzymatic activity for the InsRβ substrate in the liver from WT-NCD and WT-HFD mice treated with vehicle (WT-HFD+Veh) or Elenbecestat (WT-HFD+Ele) (n = 7, 8, 8). (**B**) Body weight of WT-NCD and WT-HFD+Veh/Ele mice during 2-month treatment (n = 8, 6, 8). (**C**) Fasting blood glucose of WT-NCD and WT-HFD+Veh/Ele mice during 2-month treatment (n = 8, 6, 8). (**D**) Body weight of WT-NCD and WT-HFD+Veh/Ele mice at the age of 8 months (at the end of 2-month Veh/Ele treatment) (n = 8, 6, 8). (**E**) Fasting blood glucose of WT-NCD and WT-HFD+Veh/Ele mice at the age of 8 months (at the end of 2-month Veh/Ele treatment) (n = 8, 6, 8). (**F**) GTTs of WT-NCD and WT-HFD+Veh/Ele mice and total glucose excursions during GTTs (n = 8, 7, 6). (**G**,**H**) ITTs of WT-NCD and WT-HFD+Veh/Ele mice (**G**) and total glucose excursions during GTTs (**H**) (n = 8, 6, 7). (**I**) Representative images of liver Oil Red O (ORO) staining (red) on the liver from WT-NCD and WT-HFD+Veh/Ele mice. Scale bar, 100 μm. (**J**) Intensity quantification of liver ORO staining (n = 6, 8, 8). (**K**) Triacylglycerol concentrations of plasma from WT-NCD and WT-HFD+Veh/Ele mice (n = 8, 8, 8). (**L**) Low-density lipoprotein concentrations of plasma WT-NCD and WT-HFD+Veh/Ele mice (n = 8, 8, 8). Data are represented as mean ± SEM. (**A**,**C**–**E**), (**F**, right), (**H**,**K**) and (**L**) One-way ANOVA followed by Tukey’s multiple comparisons test. (**B**) and (**J**) Kruskal–Wallis test followed by Dunn’s multiple comparisons test. (**F**, left) and (**G**) Mixed-effects analysis followed by Sidak’s multiple comparisons test. * *p* ˂ 0.05, ** *p* ˂ 0.01, ns means no significant differences.

To assess whether a BACE1 inhibitor can prevent diabetic phenotypes through BACE1 inhibition, WT-HFD mice were treated with one of the BACE1 inhibitors, Elenbecestat [22,23], at 10 mg/kg per day by intragastric administration for 2 months (WT-HFD+Ele). We determined that the BACE1 cleavage activity for InsRβ in the liver was significantly decreased by approximately 15% following Elenbecestat treatment (Figure 4A). WT-HFD+Ele mice showed decreased trends in body weight and fasting blood glucose levels compared to vehicle-treated HFD mice (WT-HFD+Veh) (Figure 4B–E). Meanwhile, GTT showed a decreasing trend in glucose levels and the AUC of WT-HFD+Ele mice compared to WT-HFD+Veh mice (Figure 4F). The blood glucose levels at 60 min and the AUC of ITT in the WT-HFD+Ele group were significantly decreased compared to the WT-HFD+Veh group (Figure 4G). These data indicated that Elenbecestat treatment could improve the insulin sensitivity and glucose metabolism of HFD mice.

Nonalcoholic fatty liver disease is a prevalent liver disease strongly associated with diabetes mellitus [24]. Therefore, we investigated the effect of a BACE1 inhibitor on liver fat accumulation in WT-HFD mice. We used oil red O (ORO) staining to evaluate the intracellular lipid droplet levels in liver tissue. ORO staining revealed a higher lipid accumulation in the liver tissue of the WT-HFD+Veh group than in the liver tissue of the WT-NCD+Veh group, while Elenbecestat treatment reduced hepatic lipid accumulation in the WT-HFD+Ele group (Figure 4I,J). Additionally, in terms of energy metabolism, HFD significantly increased low-density lipoprotein (LDL) and high-density lipoprotein (HDL) levels in the serum and showed a reduced trend by Elenbecestat treatment (Figure 4K,L). Thus, Elenbecestat could ameliorate diabetic phenotypes in HFD-fed mice.

### 2.4. Elenbecestat Treatment Restored Hepatic Insulin Receptor Levels and Its Downstream Pathway in HFD Mice

**Figure 5:**
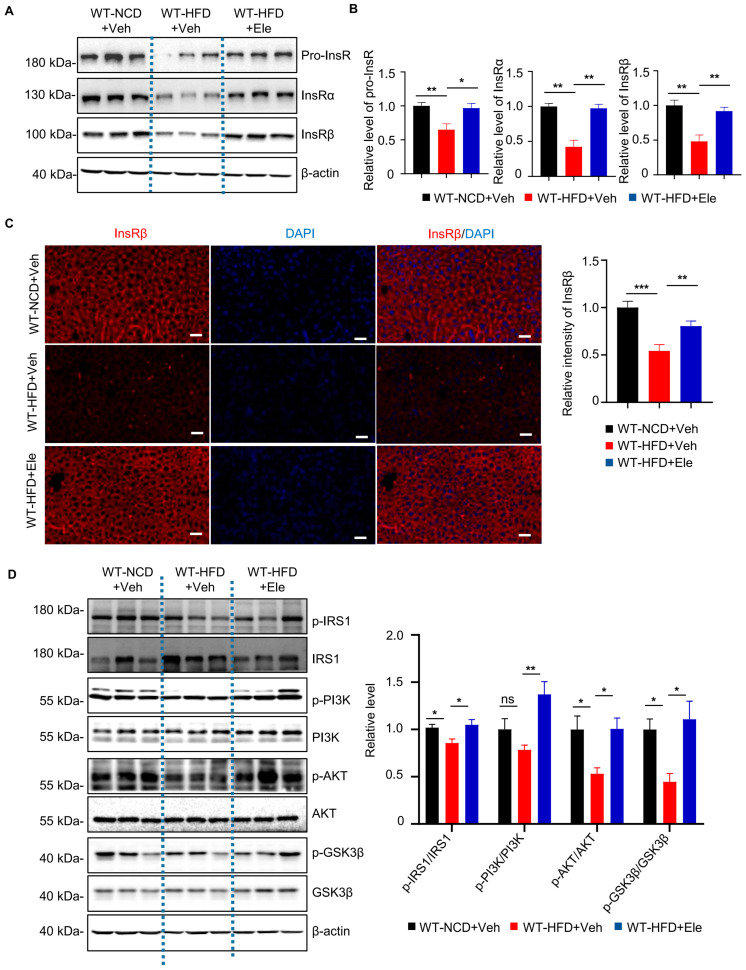
Elenbecestat treatment restored decreased InsR levels and insulin-pathway defects in the liver from HFD mice. (**A**) Western blot images showing pro-InsR, InsRa and InsRβ levels in WT-NCD and WT-HFD+Veh/Ele mice. (**B)** Statistical analysis of hepatic Pro-InsR, INSR-α, InsRβ in WT-NCD and WT-HFD+Veh/Ele mice (n = 5, 5, 5). (**C**) Representative immunostaining images of InsRβ and the statistical analysis of hepatic InsRβ in WT-NCD and WT-HFD+Veh/Ele mice (n = 8, 5, 8). Scale bar, 50 μm. (**D**) Representative western blots and statistical analysis of hepatic p-IRS1/IRS1, p-PI3K/PI3K, p-AKT/AKT and p-GSK3β/GSK3β ratio in WT-NCD and WT-HFD+Veh/Ele mice (n = 5–6). Data are represented as mean ± SEM. (**B**–**D**) One-way ANOVA followed by Tukey’s multiple comparisons test. * *p* ˂ 0.05, ** *p* ˂ 0.01, *** *p* ˂ 0.001, ns means no significant differences.

Previous research discovered that impaired insulin receptor signaling contributed to insulin resistance of diabetes mellitus [5,9], and BACE1 was identified to regulate the InsR level in HUBC mice. To explore the effects of Elenbecestat treatment on the InsR level of HFD mice, we detected the expression of full-size precursor of the InsR (pro-InsR), InsRα and InsRβ isomers in liver tissue by western blot and immunofluorescence staining. Western blot showed that levels of pro-InsR, InsRα and InsRβ (Figure 5A,B) were significantly decreased in liver tissue of WT-HFD+Veh mice compared to WT-NCD+Veh mice, which were restored in WT-HFD+Ele mice. Consistently, immunostaining showed that the InsRβ level in liver tissue was decreased in the WT-HFD+Veh mice as compared to that in WT-NCD+Veh mice, while Elenbecestat treatment could significantly increase the hepatic InsRβ level in WT-HFD+Veh mice (Figure 5C). These results indicate that treatment with Elenbecestat significantly restored hepatic InsR levels of WT-HFD mice.

After the interaction between insulin and InsR, phosphorylation of IRS1 and IRS2 occurs, activating the PI3K/Akt pathway [9,10]. Therefore, we investigated the effects of Elenbecestat on the hepatic insulin-signaling pathway in the WT-HFD mice. We found the WT-HFD mice had lower hepatic phosphorylated IRS1, AKT and GSK3β levels, as indicated by the ratios of p-IRS1/IRS1, p-AKT/AKT and p-GSK3β/GSK3β (Figure 5D), revealing that insulin signaling was disrupted in WT-HFD liver tissue. In contrast, Elenbecestat treatment significantly increased the ratio of p-IRS1/IRS1, p-PI3K/PI3K, p-AKT/AKT and p-GSK3β/GSK3β, indicating that Elenbecestat ameliorated the insulin-signaling dysfunction in the WT-HFD mice (Figure 5D). Overall, these results suggest that Elenbecestat administration could restore InsR levels and its downstream pathway.

### 2.5. Elenbecestat Treatment Ameliorated Cognitive Impairment and Anxiety Behavior in HFD Mice

**Figure 6:**
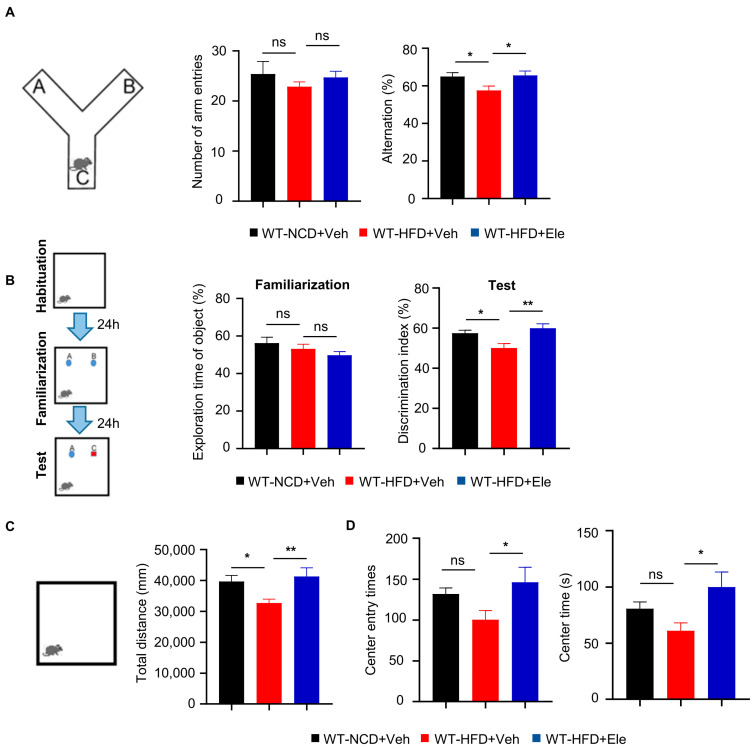
Elenbecestat treatment ameliorated cognitive impairment and anxiety behavior in HFD mice. (**A**) Arm entry times and the correct spontaneous alternations of the Y-maze test (n = 8, 7, 7). (**B**) Training discrimination index and discrimination index of the novel object recognition test (n = 7, 7, 8). (**C**) The open field test was performed to detect anxiety behavior. Total distance is shown (n = 7, 8, 7). (**D**) The open field test was performed to detect anxiety behavior. Number of entries to center and times in center is shown (n = 7, 8, 7). Data are represented as mean ± SEM. (**A**–**D**) One-way ANOVA followed by Tukey’s multiple comparisons test. * *p* ˂ 0.05, ** *p* ˂ 0.01, ns means no significant differences.

Obesity and T2DM have been linked to cognitive decline [25,26,27]. In this study, we investigated the effects of Elenbecestat on the cognitive function of HFD mice. In the spontaneous alternation Y-maze test, which assesses working memory, we observed a 15% reduction in the correction rate of spontaneously alternation in WT-HFD mice. The decline was reversed by treatment with Elenbecestat, without any significant differences noted in the total number of arm entries (Figure 6A). In the novel object recognition test, WT-HFD+Ele mice explored the novel object approximately 1.2 times longer than the WT-HFD+Veh group (Figure 6B). Moreover, previous research has indicated that obesity in mice is associated with decreased exploration and less time spent in the center during open field tests, suggesting heightened anxiety behaviors [28]. In our study, Elenbecestat-treated HFD mice showed about a 30% increase in total distance traveled, a 1.5-fold increase in the number of entries into the center and a doubling of the time spent in the center compared to vehicle-treated WT-HFD mice. This indicates that Elenbecestat may alleviate anxiety in WT-HFD mice (Figure 6C,D). Overall, these findings demonstrate that Elenbecestat treatment significantly improved cognitive impairment and reduced anxiety observed in HFD-induced obese mice.

## 3. Discussion

In this study, we observed significant increases in BACE1 level and enzyme activity in the liver of WT-HFD mice, indicating the involvement of BACE1 in obesity-induced diabetes. Meanwhile, in HUBC mice, we noted that BACE1 overexpression led to a decreased InsR level, whose downstream pathway is important in glucose homeostasis and insulin resistance. Moreover, HUBC mice exhibited increased body weight, higher blood glucose levels in GTT/ITT test and fasting condition compared to WT mice under both NCD and HFD, indicating significant insulin resistance and impaired glucose intolerance. These data suggest that BACE1-mediated InsR cleavage plays an important role in the development of T2DM.

BACE1 inhibitors have been found to induce weight loss and shown therapeutic potential for obesity, which may be beneficial for the therapy of metabolic dysfunction [29]. Elenbecestat is one of the BACE inhibitors which can reduce Aβ level in animal models and AD patients [30]. Moreover, Elenbecestat shows highest selectivity for BACE1 over BACE2, which causes minimal adverse effects [29]. The tolerated dose of Elenbecestat could be up to 300 mg/kg/day in mice according to previous studies [23], and we chose a dose of 10 mg/kg/day to minimize side effects, which was consistent with another BACE1 inhibitor MK8931 in peripheral pharmacological studies [31,32]. In this study, after a 2-month Elenbecestat treatment combined with HFD, less liver fat accumulation and decreased blood glucose levels in ITT test were found, indicating improvements in insulin resistance and glucose intolerance. Mechanically, we found that the reduced InsR level and the impaired downstream IRS1-AKT-GSK3β pathway were restored by Elenbecestat treatment, indicating the improved insulin-signaling dysfunction. Together, these results show that BACE1 inhibition improved diabetic phenotypes in WT-HFD mice through the restored InsR pathway.

There is increasing evidence that diabetes predisposes the patients to cognitive decline, leading to dementia in T2DM [33,34]. Interestingly, previous studies have shown increased BACE1 level and reduced InsR level in the brain of AD patients [18,35]. Moreover, the impairment in cerebral insulin signal would result in cognition impairment through abnormal energy metabolism and impaired neuronal activity [36,37]. These studies lead to a hypothesis that in WT-HFD mice, the elevated BACE1 may also contribute to cognition impairment through reducing the neuronal InsR level. Through the behavioral test, we observed cognition impairment in WT-HFD mice, and the Elenbecestat treatment restored cognitive ability. These results also provide a possible mechanistic explanation for the correlation between diabetes and cognition impairment, suggesting that the BACE1-InsR pathway may act as the bridge linking the two pathologies.

Taken together, our results provide further evidence of the BACE1-induced disruption on the InsR-signaling pathway in diabetes, and indicate that BACE1 is involved in the pathologies of diabetes mellitus. Furthermore, our results suggest that BACE1 inhibition could be a potential therapeutic target for diabetes mellitus through the restoration of insulin-signaling dysfunction.

## 4. Materials and Methods

### 4.1. Animals

We used 64 male C57BL/6 mice (Shanghai Experimental Animal Centre) and 18 male HUBC mice (Universal overexpression of BACE1 driven by an ubiquitin C promoter) which were generated in our laboratory and backcrossed with C57BL/6 mice for at least 8 generations. Mice were group-housed in open wire-top cages (12 hours day/night cycle, lights on at 8:00 a.m., 20–21 °C, 60–65% humidity) with ad libitum access to water and food. All animal experiments were approved by the University of Science and Technology of China (Approval Number: 202301111009000136690, date: 11 January 2023).

### 4.2. High-Fat Diet Treatments

All male C57BL/6 mice were randomly divided into the following groups at the age of 2 months for a 6-month feeding: normal chow diet (NCD; rodent diet with 10 kcal% fat, D12450J, Research Diets, New Brunswick, NJ, USA); high-fat diet (HFD; 60 kcal% fat diet, D12492, Research Diets, New Brunswick, NJ, USA). HUBC and littermate WT mice were fed with NCD and HFD at the age of 3 months for a 2-month feeding. Body weight was measured once a week, and fasting blood glucose levels were measured after fasting for 12 h.

### 4.3. Drug Treatments

After 6 months of high-fat diet treatment, WT-HFD mice were randomly divided into two groups. The WT-HFD+Ele group mice were treated with 10 mg/kg of Elenbecestat per day (Elenbecestat is dissolved in 0.5% carboxymethylcellulose sodium) for 2 months through intragastric administration. The WT-HFD+Veh group were given vehicle (0.5% carboxymethylcellulose sodium) for 2 months through intragastric administration. After 6 months of NCD treatment, the WT-NCD+Veh mice were also given vehicle (0.5% carboxymethylcellulose sodium) as the control group. After 2 months of drug treatment, tissues were collected and experiments were carried out.

### 4.4. Metabolic Measurements

Glucose tolerance test (GTT) and insulin tolerance test (ITT) were performed at the end of months 6 (WT/HUBC-NCD mice), 8 (WT-NCD/HFD and WT/HUBC-HFD mice) and 10 (WT-NCD and WT-NCD+Veh/Ele mice). All mice were fasted overnight (10 h) before GTT and 4 h before ITT. Tail blood glucose was determined at 0 min (before intraperitoneal) injection of 1 g/kg glucose (G5500, Sigma-Aldrich, St. Louis, MO, USA) or 0.5 U/kg insulin (1342106, Sigma-Aldrich, St. Louis, MO, USA) and 15, 30, 60 and 90 min post-injection.

### 4.5. Western Blotting

Mice fasted for 12 h were anesthetized by intraperitoneal injection of tribromo-ethanol, and the tissues were dissected and stored at ×80 °C. The tissues were homogenized in lysis buffer (50 mM Tris (T6687, Sigma, St. Louis, MO, USA), 150 mM NaCl (S5886, Sigma-Aldrich, St. Louis, MO, USA), 1% NP-40 (A600385, Sangon Biotech, Shanghai, China), 0.5% sodium deoxycholate (D6750, Sigma-Aldrich, St. Louis, MO, USA); the homogenates were ultrasonically broken for 3 min and centrifuged (16,000× *g*, 4 °C, 40 min). Supernatants were collected and protein concentrations were quantified by Bicinchonic Acid Protein Assay Kit (23225, Thermo Scientific, Waltham, MA, USA), and the final protein concentration was adjusted to 5 μg/μL in lysis buffer. The protein samples were separated by 10–12% SDS-PAGE gels and transferred onto 0.45 μm nitrocellulose membranes. The membranes were blocked in 5% milk (1706404, Bio-Rad, Hercules, CA, USA) at room temperature for 1 h, and incubated overnight with primary antibodies: rabbit anti-BACE1 antibody (5606S, Cell signaling technology, Danvers, MA, USA), rabbit anti-Insulin receptor α antibody (74118S, Cell signaling technology, Danvers, MA, USA), rabbit anti-Insulin receptor β antibody (3025S, Cell signaling technology, Danvers, MA, USA), rabbit anti-IRS (95816S, Cell signaling technology, Danvers, MA, USA), rabbit anti-phosphor-IRS (ser612) (2386S, Cell signaling technology, Danvers, MA, USA), rabbit anti-AKT (9272, Cell signaling technology, Danvers, MA, USA), rabbit anti-phosphor-AKT (9271, Cell signaling technology, Danvers, MA, USA), rabbit anti-PI3K (11889S, Cell signaling technology, Danvers, MA, USA), rabbit anti-phosphor-PI3K (4228S, Cell signaling technology, Danvers, MA, USA), rabbit anti-GS3Kβ (12456S, Cell signaling technology, Danvers, MA, USA), rabbit anti- phosphor-GSK3β (5558S, Cell signaling technology, Danvers, MA, USA), mouse anti-β-actin (4970S, Cell signaling technology, Danvers, MA, USA) at 4 °C. Then, the membranes were washed 5 times with TBS (B548105, Sangon Biotech, Shanghai, China) containing 0.1% Tween-20 (9005-64-5, Sangon Biotech, Shanghai, China), and incubated in Goat-anti-Rabbit IgG secondary antibody (7074, Cell signaling technology, Danvers, MA, USA) at room temperature for 90 min; then, the membranes were washed 5 times with TBS containing 0.1% Tween-20, and 3 times with TBS. proteins were visualized using enhanced chemiluminescent substrate and images were captured with an ultrasensitive chemiluminescence imager. Protein levels were quantified via ImageJ software, version 1.51J8.

### 4.6. Oil Red O (ORO) Staining

Liver tissues were fixed with 4% paraformaldehyde (PFA) (158127, Sigma-Aldrich, St. Louis, MO, USA) for 24 h, and then dehydrated with sucrose gradient (A100335, Sangon Biotech, Shanghai, China) for 24 h, respectively. Next, the liver tissues were cut to 25 μm thickness. After being washed for 5 min with 1× PBS (B548117, Sangon Biotech, Shanghai, China), the liver tissues were stained with ORO solution (C0158S, Beyotime, Shanghai, China) for 15 min. Images were analyzed using ImageJ software, version 1.51J8.

### 4.7. BACE1 Enzyme Activity Assay

BACE1 activity assays were performed as previously described. Synthetic InsR and APP^sw^ peptide substrates containing BACE1 cleavage sites (MCA-Trp-Thr-Glu-Pro-Thr-Tyr-Phe-Tyr-Val-Thr-Asp-Lys(Dnp)-Arg-Arg-NH2 and MCA-Ser-Glu-Ile-Asp-Leu-Met-Val-Leu-Asp-Arg-Lys(Dnp)-Arg-Arg-NH2, respectively) were dissolved in 2 mM stock solution with dimethyl sulfoxide (DMSO) (D8418, Sigma-Aldrich, St. Louis, MO, USA), and then diluted in the reaction buffer (50 mM sodium acetate (B300606, Sangon Biotech, Shanghai, China), pH 3.5). The liver tissue was homogenized in a lysis buffer (10 mM Tris-HCl (1185-53-1, Sigma-Aldrich, St. Louis, MO, USA), 150 mM NaCl, 1 mM EDTA (PHR2586, Sigma-Aldrich, St. Louis, MO, USA), 1 mM EGTA (E3889, Sigma-Aldrich, St. Louis, MO, USA), 1 mM Na3VO4 (20040526, MedChemExpress, Monmouth Junction, NJ, USA), 10% glycerol (V900502, Sigma-Aldrich, St. Louis, MO, USA), 0.5% Triton X-100 (V900502, Sigma-Aldrich, St. Louis, MO, USA), pH 7.4). The homogenates were ultrasonically broken for 3 min and centrifuged (16,000× *g*, 4 °C, 40 min). Supernatants were collected and protein concentrations were quantified by BCA kit, and the final protein concentration was adjusted to 9 mg/mL in lysis buffer. The protein samples were diluted in the reaction buffer; mice’s blood was collected using centrifuge tubes containing anticoagulant and centrifuged (2000× *g*, 10 min, 4 °C), plasma was collected and apportioned into 50 μL aliquots and stored at −80 °C. The plasma was diluted with reaction buffer. A 100 μL reaction system (EX/EM, 320 nm/405 nm) containing diluted samples and substrates was used to investigate BACE1 enzyme activity by using a micro plate reader (SynergyH1, Biotek, Winooski, VT, USA).

### 4.8. Immunofluorescence Staining

Paraffin-embedded liver tissue was cut to 4 μm sections and kept at 60 °C for 1 h in the oven and then deparaffinized with xylene for 15 min and hydrated with an ethanol gradient (100%, 95%, 85%, 75%, 50%) for 5 min, respectively. Antigen retrieval was per-formed at the condition of 100 °C in a thermostat water bath with ethylene diamine tetraacetic acid (EDTA, pH 9.0). Then, the tissues were washed 2 times with 1xPBS and incubated with 0.5% Triton x-100 at room temperature for 1 h. Next, tissues were washed 5 min in PBS and incubated with 5% normal goat serum (NGS) (C0265, Beyotime, Shanghai, China) at room temperature for 1 h. Tissues were washed 5 min in PBS and incubated overnight with primary antibody (rabbit anti-insulin receptor β (23413s, Cell signaling technology, Danvers, MA, USA) at 4 °C. Tissues were washed 5 times with PBS, and incubated with Anti-rabbit Alexa Fluor^®^ 555 secondary antibody (A-11004, Invitrogen, Carlsbad, CA, USA) containing DAPI (4083, Cell signaling technology, Danvers, MA, USA) for 1 h at room temperature and washed 5 times in PBS. Images were taken using a fluorescence microscope (TissueFAXS CHROMA, TissueGnostics, Vienna, Austria) and analyzed using Image J software.

### 4.9. Serum Biochemical Analysis

At the end of all the experiments, after 12 h of fasting, blood was collected from mice. After standing at 4 °C for 2 h, the serum was isolated by centrifugation at 2000 rpm for 15 min. Measurement of low-density lipoprotein (LDL) and triglycerides (TG) were con-ducted by Thermo Scientific™ Indiko™ Automatic Biochemical Analyzer (Mindrite BS-420, Thermo Scientific, Waltham, MA, USA). The kits used in the experiments were purchased from Zhongsheng Beikong Biotechnology Co., Ltd. (Beijing, China).

### 4.10. Behavior Tests

For the open field test, following a 2 h acclimation, mice were individually placed in a polypropylene arena (60 cm × 160 cm, 50 cm high walls) under dim lighting conditions for a 10 min session. Locomotor activity (total travel distance and average speed) was quantified using an automated video-tracking system.

For novel object recognition test, mice were first allowed to freely explore two identical objects for 10 min in a dimly lit open field arena. After a 2 h retention interval, one object was replaced with a novel object, and exploration behavior was recorded for another 10 min. Recognition memory was evaluated by calculating the recognition index, defined as the ratio of time spent investigating the novel object to the total time spent exploring both objects. Manual video scoring was employed for behavioral analysis.

For the Y-maze test, spatial working memory and spontaneous exploration were assessed in a Y-shaped maze (30 cm arm length × 13 cm width, 30 cm wall height). Mice were positioned in the central zone, and arm entries were recorded over a 3 min trial. The working memory index was calculated as the percentage of spontaneous alternations (sequential entries into three distinct arms) relative to total arm entries, while total entries served as a measure of exploratory drive.

### 4.11. Statistical Analysis

All data are presented as mean ± SEM. The differences were claimed significant when *p* < 0.05. For two groups, the unpaired *t* test with Welch’s correction was used for normally distributed data and the Mann–Whitney test for non-normally distributed data. For three groups, one-way ANOVA followed by Tukey’s multiple comparisons was used to analyze the normally distributed data and the Kruskal–Wallis test followed by Dunn’s multiple comparisons test was used to analyze the non-equal variances or non-normally distributed data. Two curves were compared by mixed-effects analysis followed by Sidak’s multiple comparisons test. Statistical analyses were performed using GraphPad 8.0 software.

## Data Availability

The original contributions presented in this study are included in the article. Further inquiries can be directed to the corresponding authors.

## Abbreviations

The following abbreviations are used in this manuscript:

BACE1	Β-secretase 1
AD	Alzheimer’s disease
T2DM	Type 2 diabetes mellitus
HFD	High-fat diet
HUBC	Systemic overexpression of bace1
InsR	Insulin receptor
App	Amyloid precursor protein
WT	Wild-type
Ele	Elenbecestat
NCD	Normal chow diet
GTT	Glucose tolerance test
ITT	Insulin tolerance test
ORO	Oil Red O
BCA	Bicinchonic acid
NGS	Normal goat serum
LDL	Low-density lipoprotein
TG	Triglycerides
AUC	Area under the curve
WAT	White adipose tissue
ns	No significant differences
HDL	High-density lipoprotein
